# CT Imaging Findings after Stereotactic Radiotherapy for Liver Tumors

**DOI:** 10.1155/2015/126245

**Published:** 2015-06-29

**Authors:** Olga R. Brook, Eavan Thornton, Mishal Mendiratta-Lala, Anand Mahadevan, Vassilious Raptopoulos, Alexander Brook, Robert Najarian, Robert Sheiman, Bettina Siewert

**Affiliations:** ^1^Department of Radiology, Beth Israel Deaconess Medical Center, 330 Brookline Avenue, Boston, MA 02215, USA; ^2^Department of Radiology, Beaumont Hospital, Dublin, Ireland; ^3^Henry Ford Hospital, Diagnostic Radiology, 2799 West Grand Boulevard, Detroit, MI 48202, USA; ^4^Department of Radiation Oncology, Beth Israel Deaconess Medical Center, 330 Brookline Avenue, Boston, MA 02215, USA; ^5^Department of Pathology, Beth Israel Deaconess Medical Center, 330 Brookline Avenue, Boston, MA 02215, USA

## Abstract

*Purpose*. To study radiological response to stereotactic radiotherapy for focal liver tumors. *Materials and Methods*. In this IRB-approved, HIPAA-compliant study CTs of 68 consecutive patients who underwent stereotactic radiotherapy for liver tumors between 01/2006 and 01/2010 were retrospectively reviewed. Two independent reviewers evaluated lesion volume and enhancement pattern of the lesion and of juxtaposed liver parenchyma. *Results*. 36 subjects with hepatocellular carcinoma (HCC), 25 with liver metastases, and seven with cholangiocarcinoma (CCC) were included in study. Mean follow-up time was 5.6 ± 7.1 months for HCC, 6.4 ± 5.1 months for metastases, and 10.1 ± 4.8 months for the CCC. Complete response was seen in 4/36 (11.1%) HCCs and 1/25 (4%) metastases. Partial response (>30% decrease in long diameter) was seen in 25/36 (69%) HCCs, 14/25 (58%) metastases, and 7/7 (100%) of CCCs. Partial response followed by local recurrence (>20% increase in long diameter from nadir) occurred in 2/36 (6%) HCCs and 4/25 (17%) metastases. Liver parenchyma adjacent to the lesion demonstrated a prominent halo of delayed enhancement in 27/36 (78%) of HCCs, 19/21 (91%) of metastases, and 7/7 (100%) of CCCs. *Conclusion*. Sustainable radiological partial response to stereotactic radiotherapy is most frequent outcome seen in liver lesions. Prominent halo of delayed enhancement of the adjacent liver is frequent finding.

## 1. Introduction

Stereotactic radiotherapy allows precise delivery of ablative radiation dose to a tumor, sparing the surrounding structures. Initially it was used for treatment of brain lesions with very high local control [1]. Its use in abdominal and pelvic organs was limited due to respiratory motion; however, new systems were developed using real-time tracking of respiration with adjustment of the treatment beam to overcome motion.

About 80% of patients diagnosed with liver tumors are not eligible for definitive surgical treatment due to comorbidities and extent of the disease [2, 3]. Multiple options are available for treating unresectable tumors, such as chemotherapy, transarterial chemoembolization, Y90 microsphere embolization, and radiofrequency (or other types of) ablation. Stereotactic radiotherapy has been introduced as an additional treatment option, as all the preceding therapies have various limitations depending on tumor size, location, number, and distribution. Stereotactic radiotherapy has already been shown to be an effective and safe treatment for hepatocellular carcinoma [4, 5] and hepatic metastases [6–10]. The benefits of stereotactic radiotherapy include the ability to treat tumors that are difficult to access percutaneously, sparing of normal parenchyma adjacent to the tumor [11], and delivery of therapy in a single or few (less than five) treatment sessions. Some authors suggest inclusion of stereotactic radiotherapy in the treatment guidelines of hepatic tumors [12].

It is important to know the expected appearance of a lesion after stereotactic radiotherapy in order to be able to differentiate it from local recurrence or progression of disease. Radiological appearances of liver lesions and adjacent liver tissue after stereotactic radiotherapy have not been specifically studied, apart from short mention in a number of studies that have evaluated efficacy of the stereotactic radiotherapy [8]. In this study we determine the changes expected on cross-sectional imaging at different times after treatment of a variety of malignant liver lesions.

## 2. Material and Methods

### 2.1. IRB

The study was conducted with the approval of our institutional review board and was compliant with Health Insurance Portability and Accountability Act regulations. Informed consent was waived by the institutional review board due to the retrospective nature of the study.

### 2.2. Patients

All patients with liver lesions that were treated with stereotactic radiotherapy at our institution between January 2006 and January 2010 were identified from our stereotactic radiotherapy database and included in this study. All cases were presented at the weekly multidisciplinary liver conference at which oncologists, gastroenterologists, surgeons, diagnostic and interventional radiologists, and pathologists review the cases collectively to decide upon optimal treatment. The focal liver lesions that are usually referred for stereotactic radiotherapy are those that are not surgical candidates and not appropriate candidates for radiofrequency ablation (lesions larger than 3 cm; adjacent to the vital structures, such as colon, small bowel, stomach, gallbladder, and common bile duct; or without safe percutaneous access).

### 2.3. Imaging Studies

For baseline, we used the CT exam carried out at the simulation radiotherapy session performed within 10 days prior to the onset of treatment. If a simulation study was performed without intravenous contrast then a contrast enhanced CT performed within one month prior to onset of treatment was used as a baseline study. For each subject, every posttherapy contrast-enhanced CT study performed up to the end of our study period (1/2010) was evaluated.

The simulator radiotherapy CT consisted of a portal venous phase only scan performed on a Toshiba 64-row MDCT scanner (Toshiba America Medical Systems, Tustin, CA) at 120 kVp, 200 mA, reconstructed with 2.5 mm slice width and 2.5 mm interval. 130 cc of intravenous contrast (Optiray 320, (320 mg/mL)) via an antecubital vein at 3-4 cc/second was administered and scanning started 70 sec after initiation of the injection.

All patients were treated with respiratory motion tracking image-guided radiotherapy (Synchrony (Accuray Incorporated, Sunnyvale, CA)). Patients received radiation therapy in 1 to 5 fractions with target lesion coverage above 90%. We have not identified any cases of acute or late toxicity. Child Pugh Scores (CPS) were documented and tracked by the hematologists and oncologists. While there was no acute treatment related deterioration in CPS, most patients had their preexisting long term decline in their CPS.

Follow-up CT and baseline formal CT studies were performed on GE 64-detectors MDCT scanners (VCT or HD 750, General Electric Medical Systems, Milwaukee, WI). The imaging protocol included low dose noncontrast scan using 120 kVp, 50–150 mA followed by IV contrast enhanced scans. Portal venous phase scan was done for metastatic disease and multiphase scan for HCC and CCC. In our multiphase protocol, scanning was done in the late arterial, portal venous, and equilibrium 3-minute delay phase for HCC and 10 min delay in CCC, all with 120 kVp, automatic tube modulation, reconstructed at 2.5 mm width with 2.5 mm interval. For the multiphase scans, 150 cc of contrast medium (Omnipaque 350, (350 mg/mL)) was administered at a rate of 4 cc/sec and continuous tracking of contrast enhancement in the aorta was used to trigger scanning using a 280 HU threshold for the late arterial phase (around 40 sec), followed 30 seconds later with a portal venous phase scan (around 70 sec). In the liver metastasis cases following low mA noncontrast scan, 130 cc of IV contrast was injected at a rate of 2.5–3.5 cc/sec and portal venous scan was done at 70 sec followed by 3 min delay equilibrium scan.

### 2.4. Image Evaluation

The three largest perpendicular dimensions (anterior-posterior, craniocaudal, and right-left) of the targeted lesion were measured on axial and coronal images on the portal venous phase on all studies. If more than one lesion was treated in the same radiotherapy session, then all treated lesions were included in the study. The lesion's dimensions were measured on PACS station (GE Medical, Milwaukee, WI) independently by two reviewers, with 7 and 5 years of experience in abdominal imaging, respectively. The volume of the lesion on all studies was calculated according to the ellipsoid formula: product of anterioposterior, transverse, and craniocaudal dimensions multiplied by *π*/6.

Quantification of the amount of enhancement within the lesion in the arterial phase on the follow-up was subjectively graded 0 to 5 by two reviewers: 0 corresponds to 100% enhancement, 1 corresponds to 75–99% of enhancement, 2 corresponds to 50–74% of enhancement, 3 corresponds to 25–49% of enhancement, 4 corresponds to 1–24% of enhancement, and 5 corresponds to no enhancement.

Lesion response was further classified from the diameter measurements of the enhancing portion, following RECIST guidelines for hepatocellular carcinoma [13]: complete response defined as disappearance of any intratumoral arterial enhancement and partial response as at least 30% decrease in the sum of diameters of the enhancing portion of the lesion (using the baseline sum of the diameters of the lesion as reference); response followed by recurrence as increase of at least 20% in the sum of the diameters of the enhancing portion of the lesion, after initial partial response as described above. Response for cholangiocarcinoma and metastases was assessed in the similar manner.

Further, the liver parenchyma around the treated lesion was classified as hypodense, isodense, or hyperdense to the rest of the liver on the arterial, portal venous, and delayed phases. Under this characterization we excluded cases with focal/nodular appearance of the enhancement or change in the enhancement (such as wash out) at the delayed phase.

### 2.5. Statistical Analysis

Means with 95% confidence intervals, medians, standard deviation, and range were used to describe clinical and radiological characteristics of the study groups. Wilcoxon signed rank test was used to compare appearance of the liver parenchyma adjacent to the treated lesion prior to and after treatment. Paired Student's *t*-test was used to compare percentage of unenhanced area and lesion volumes prior to and after treatment. Interobserver variability was evaluated by linear weighted *κ* statistics, with weights proportional to the difference in grades. Statistical significance was set at *p* = 0.05.

## 3. Results

### 3.1. Hepatocellular Carcinoma Group

Overall response grading had excellent agreement between the two observers (*κ* = 0.99). The HCC group included 32 patients with 36 hepatocellular carcinoma lesions. The patients were treated with mean total dose of 3189 ± 772 cGy (range 2000–4500 cGy), in median 3 (range 1–5) fractions with mean dose of 1058 ± 429 cGy (range 450–2400 cGy) per fraction. The mean lesion volume was 15346 ± 89083 cc (range 6–527306 cc, median 80 cc). The mean lesion coverage was 95 ± 1% (range 93–98%). The average prescription isodose was 78 ± 5% (range 66–86%). All subjects had at least one lesion with pathologic confirmation. In the cases with multiple lesions, if the imaging characteristics of the additional lesions were similar to their confirmed tumor, then the other lesions were assumed to have the same pathology (Table 1). All patients had liver cirrhosis. One patient was previously treated with RFA and two other patients have been previously treated with chemotherapy. No patients were treated with chemotherapy during stereotactic radiotherapy treatments. Patients clinical characteristics are described in Table 1. Complete response was seen in 4 lesions (11%), partial response in 25 lesions (69%), and partial response followed by local recurrence in 2 (6%). Complete response was seen in four lesions, with follow-up of 3, 12, 12, and 24 months, respectively. Recurrence occurred in 2 patients two and three months after treatment, respectively. The average decrease in lesion volume per month of follow-up was 24.5% (95% CI, 11.9–37.1) in the first 4 months, 9.8% per month (95% CI, 3.6–15.6), four to nine months after treatment, and 2.7% per month (95% CI, 0.7–4.7) thereafter. The difference between pretreatment and posttreatment volumes of the treated lesion was statistically significant (*p* < 0.001). The dynamics of the volume changes over the follow-up time can be seen in Figure 2. The average nonenhancing portion prior to treatment was 27 ± 37% and 59 ± 33% (*p* = 0.002) after treatment. Lack of enhancement within the lesion was seen usually on the first CT study performed between 15 to 45 days after treatment and persisted on the consequent studies, unless a recurrence occurred (Figure 3).

During follow-up the appearance of the liver parenchyma surrounding the treated lesion changed from isodense to relatively higher attenuation in 27/36 lesions (75%). In these cases, high attenuation of the adjacent liver parenchyma was seen on all postcontrast phases and persisted on all follow-up studies. The liver appeared isodense to the rest of the liver in 8/36 lesions (22%) and hypodense in 1/36 lesions (3%), as seen in Figure 4. The appearance of the liver parenchyma after treatment was significantly different from before treatment (*p* < 0.001).

In two cases, patients underwent liver transplantation (6 and 7 months after stereotactic radiotherapy). The pathology of the liver explants in the liver adjacent to the treated lesion showed fibrosis with embedded bile ductules, regenerating hepatocytes, and fibroblasts indicative of prior ablative therapy (Figure 5). In these cases liver parenchyma adjacent to the target lesions had high attenuation on the portal venous and delayed phases.

### 3.2. Metastases Group

There were 20 patients with 24 metastases treated in the study. The metastases were from colon cancer in 7 patients, melanoma in 4 patients, lung, breast, and pancreas carcinoma in 2 patients each, and renal, duodenal carcinomas, and carcinoid in one patient each. Patients clinical characteristics are described in Table 1. The patients were treated with mean total dose of 3400 ± 717 cGy (range 2400–4500 cGy), in median 3 (range 1–5) fractions with mean dose of 1183 ± 362 cGy (range 600–2400 cGy) per fraction. The mean lesion volume was 127  ±  152 cc, median 80 cc (range 4–524 cc). The mean lesion coverage was 95  ±  2% (range 90–99%). The average prescription isodose was 77  ±  4% (range 66–84%). All patients were previously treated with chemotherapy; however none were treated with chemotherapy during the stereotactic radiotherapy treatment. Complete response was seen in 1/25 lesions (4%), partial response in 14/25 lesions (58%), and partial response followed by local recurrence in 4/25 lesions (17%). For the patients with either complete response or partial response there was a significant difference between the volume prior to and after treatment (*p* = 0.02). The dynamics of the volume changes over the follow-up time can be seen in Figure 6. The area without enhancement within the treated metastatic lesion rose on the first follow-up study (15–45 days after treatment) and in most of the cases did not change significantly over the follow-up period unless a recurrence occurred (Figure 7). The average area without enhancement prior to treatment was 20 ± 35% and after treatment 61 ± 37% (*p* < 0.001).

Liver surrounding the treated lesion was hyperdense to relative adjacent normal liver in 21 lesions (88%) and isodense to the surrounding liver in 3 lesions (12%), with examples shown in Figure 8. In all cases prior to treatment the liver around the lesion was isodense to the rest of the liver parenchyma. High attenuation of the adjacent liver parenchyma was seen on all postcontrast phases but was most prominent at the delayed phase. Appearance of the liver parenchyma after treatment was significantly different from before treatment (*p* < 0.001). Hypodense halo around the lesion was seen initially in 3/24 cases on the first follow-up (3 or less months after treatment), with change to hyperdense halo on further follow-up studies (Figure 9). If high attenuation of adjacent liver parenchyma was noted, then it was present on all follow-up studies.

### 3.3. Cholangiocarcinoma Group

There were 7 patients each with a single lesion. Patients clinical characteristics are described in Table 1. The patients were treated with mean total dose of 3100 ± 727 cGy (range 2400–4500 cGy), in 3 fractions with mean dose of 1033 ± 242 cGy (range 800–1500 cGy) per fraction. The mean lesion volume was 130 ± 110 cc, median 130 cc (range 21–234 cc). The mean lesion coverage was 96 ± 2% (range 94–98%). The average prescription isodose was 74 ± 4% (range 70–79%). Two patients were previously treated with chemotherapy, 1 patient with TACE, and 1 patient with RFA. During stereotactic radiotherapy patients were not treated with chemotherapy. Partial response was seen in all 7/7 patients (100%). The area without enhancement within the lesion was present in only 2/7 cases (29%). The difference between pretreatment and posttreatment volumes of the treated lesion was statistically significant (*p* = 0.05). The dynamics of the volume changes over the follow-up time can be seen in Figure 10. In all cases the liver surrounding the treated lesion was hyperdense on the portal venous phase (Figure 11). In all cases prior to treatment the liver around the lesion was isodense to the rest of the liver parenchyma. High attenuation of the adjacent liver parenchyma was seen on all postcontrast phases but was most prominent at the delayed phase and was seen on all follow-up studies. The appearance of the liver parenchyma after treatment was significantly different from before treatment (*p* < 0.001).

We have not identified any case of acute or late toxicity due to stereotactic radiotherapy.

## 4. Discussion

**Figure 1:**
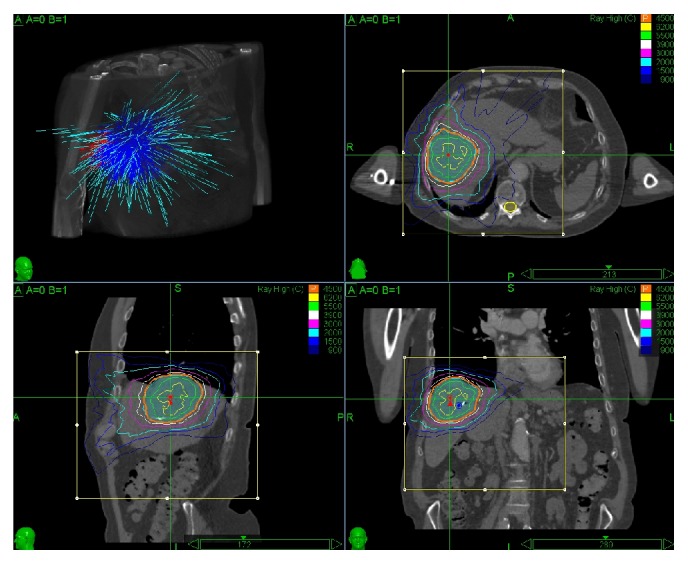
Image capture from stereotactic radiotherapy planning session, showing concentration of radiation dose to the tumor with much smaller but still significant amount of radiation delivered to the surrounding liver.

**Figure 2:**
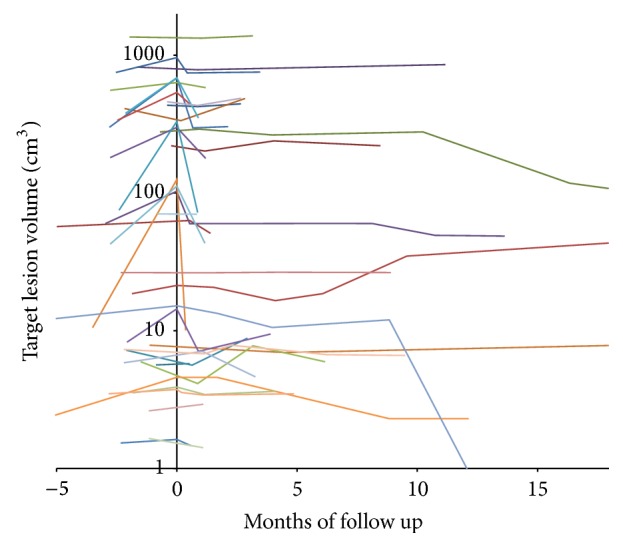
Target lesion volume changes in hepatocellular carcinoma patients (each curve denotes an individual lesion). Most of the lesions show volume decrease most prominently soon after treatment, with some lesions demonstrating further slower volume decrease with time. In a few cases there was an increase in volume later on, corresponding to recurrence.

**Figure 3:**
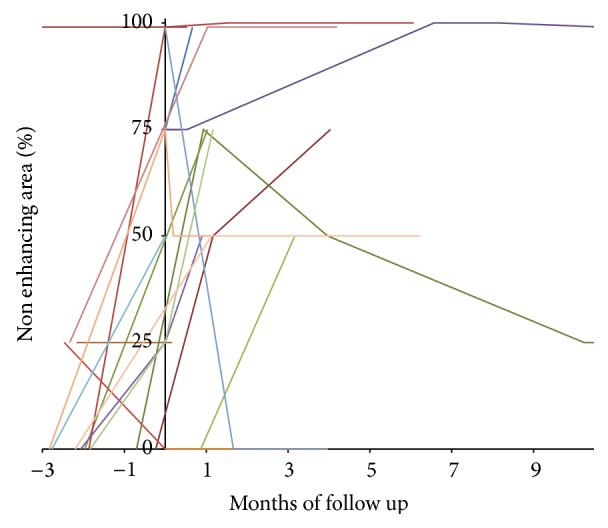
Nonenhancing areas within the hepatocellular carcinoma lesions over the follow-up period. Most of the lesions demonstrate increase in the nonenhancing area after treatment, mostly without significant change later on, unless a recurrence occurs.

**Figure 4:**
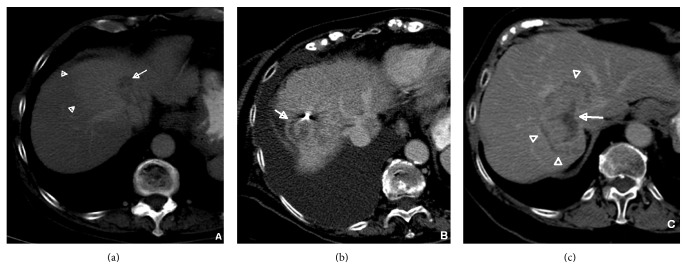
Three types of responses of surrounding liver parenchyma to stereotactic radiotherapy treatment of hepatocellular carcinoma lesions, as seen on delayed imaging: hyperdense halo (a), the most frequent response, followed by isodense liver parenchyma (b), and hypodense halo (c). Arrows are pointing to the treated lesion and arrowheads to the response of the surrounding liver.

**Figure 5:**
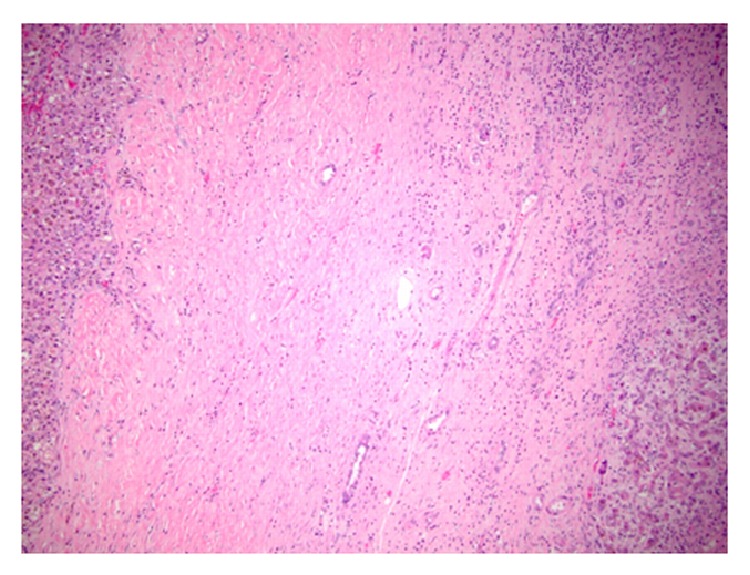
Liver explants histopathology showing fibrosis with embedded bile ductules, regenerating hepatocytes, and fibroblasts indicative of prior radiation therapy.

**Figure 6:**
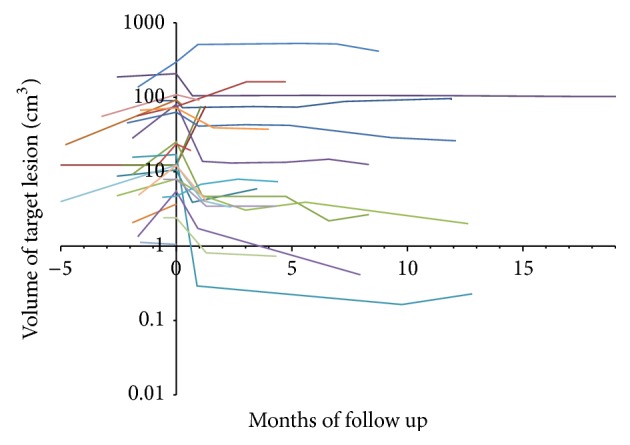
Target lesion volume changes in patients with metastases (each curve denotes an individual lesion). Most of the lesions show decrease in volume soon after treatment, followed by some additional decrease in volume, unless a recurrence occurs.

**Figure 7:**
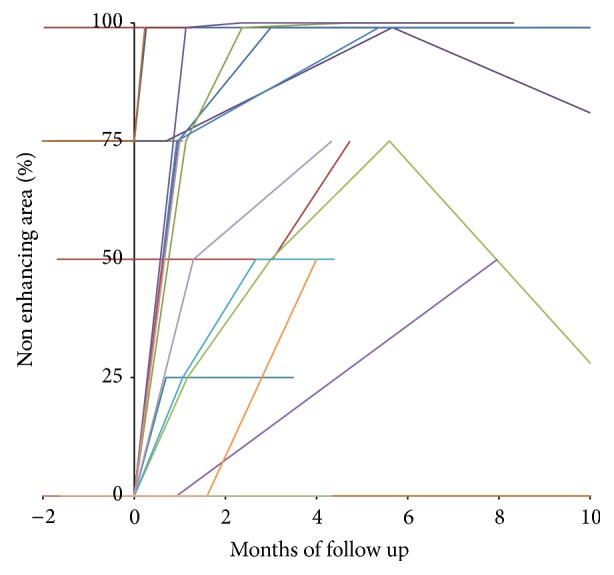
Nonenhancing area within the treated metastatic lesion over the follow-up period (each line represents an individual lesion).

**Figure 8:**
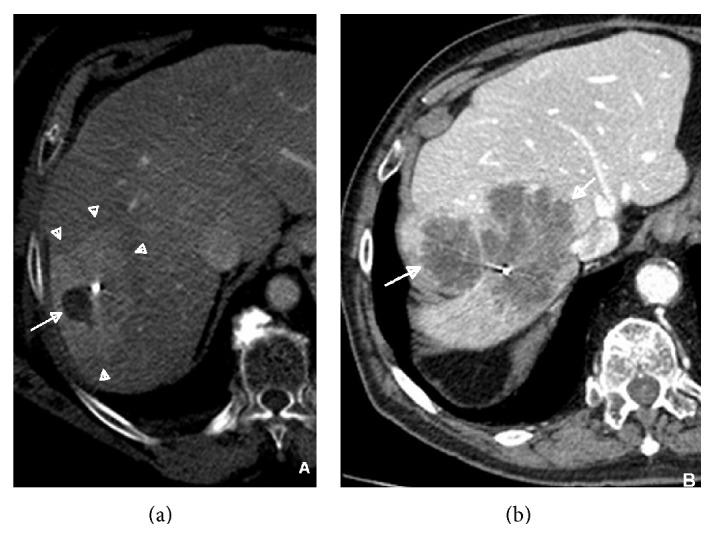
Two patterns of response in the surrounding liver parenchyma following treatment of metastases, the most prevalent being hyperdense halo (a) and isodense to the rest of the liver (b). Arrows point to the treated lesion and arrowheads to the response of the surrounding liver.

**Figure 9:**
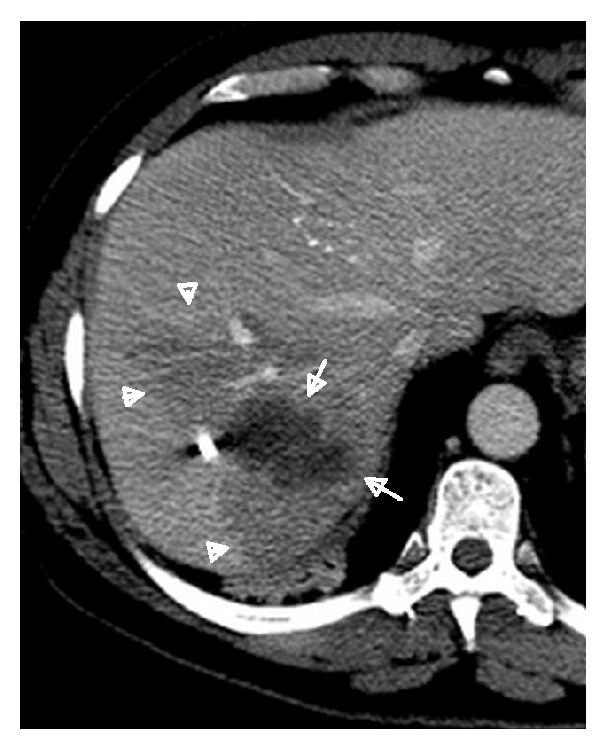
49-year-old female patient with melanoma. In the CT scan performed 3 months after treatment a hypodensity (arrowheads) around the treated lesion (arrows) is seen. Of note, a radiopaque fiducial seed is seen laterally to the lesion.

**Figure 10:**
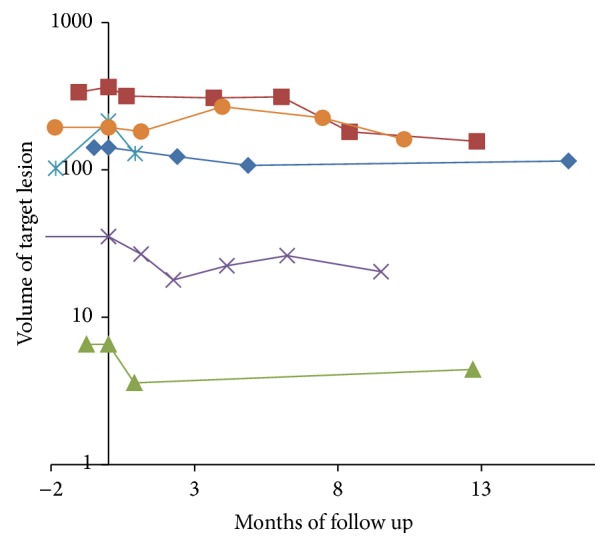
Target lesion volume changes in patients with cholangiocarcinoma (each curve denotes an individual lesion). All lesions continuously decreased in volume after treatment, initially at a fast rate which decreased over time. In all cases there was a residual lesion left, representing a partial response.

**Figure 11:**
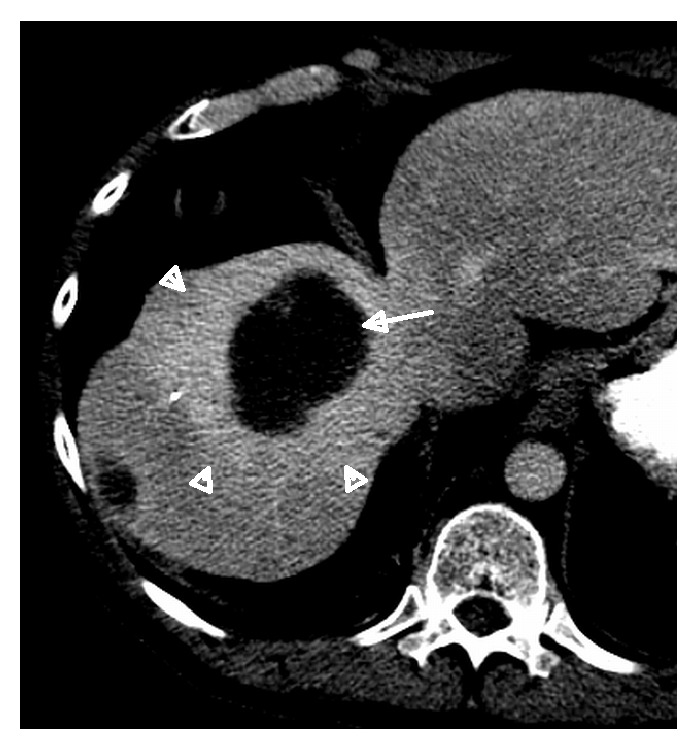
56-year-old male patient with cholangiocarcinoma. Axial CT at delayed phase showing hypodense treated cholangiocarcinoma without residual enhancement (arrow), accompanied by halo of hyperdensity (arrow heads).

Stereotactic radiotherapy is considered an effective palliative treatment for HCC, metastases, and cholangiocarcinoma [4–9, 14]. In our study population of primary HCC, cholangiocarcinoma, and metastatic disease, the most frequent pattern of response was a decrease in lesion volume that was most pronounced in the first four months, followed by continuous but slower decrease in lesion volume. The nonenhancing portion of the lesion increased to a maximum within the first 3 months after treatment and did not change significantly over the follow-up period unless a recurrence occurred. In rare cases a complete response of a lesion can occur (11% in HCC and 4% of cholangiocarcinoma). Our clinical results from stereotactic radiotherapy are in agreement with previously published phase I/II studies [15, 16] and more recent small series of 25 patients with HCC [5] and 17 patients with variety of liver lesions [4] in which local control was seen in 82–95% of cases, as well as with larger more recent series [17–19]. We have not observed a significant liver toxicity, similar to the prior studies [20–22]. A striking feature that we noted in the majority of the cases was a halo of hyperdensity in the liver surrounding the treated lesion on the portal venous and delayed phases. In two cases of hepatocellular carcinoma, a histopathological correlate was available, with explants evaluated 6 and 7 months after the radiotherapy treatment. The pathology in the areas adjacent to the treated lesions showed findings of fibrosis consistent with postradiation treatment changes. Fibrosis is known to show progressive enhancement that peaks on the delayed/portal venous phase, for example, in the case of confluent hepatic fibrosis [23]. As can be seen in a stereotactic radiotherapy planning session (Figure 1), the liver surrounding the treated lesion is also irradiated, albeit with a smaller dose. Radiation induced injury to the liver has been described previously. As early as in 1965, Ingold et al. [24] reported radiation hepatitis in a cohort of patients treated for gynecological malignancy with whole abdomen external beam radiation. Further studies with histopathologic correlation [25, 26] showed that radiation-induced liver disease has two phases: acute phase occurring within 3 months after exposure with sinusoidal congestion, hyperemia, and diffuse fatty infiltration corresponding to hypodensity of the surrounding liver as seen in our cases [27]. As shown in a histopathological study by Lewin and Millis [28], portal tracts fibrosis and disorganization of the lobular architecture without sinusoidal congestion occur in a later chronic phase. In our cohort this corresponded to enhancement on the delayed/portal venous phase images, which is typical for fibrosis. The differentiation of types of enhancement abutting a lesion is important as a diffuse delayed phase enhancement is more likely a response to radiation fibrosis and should not be misinterpreted as locally recurrent tumor enhancement.

Our study has several limitations. First, the retrospective nature of our study resulted in varying length and frequency of follow-up and variability of the CT study technique (phases of imaging). Secondly, histopathological correlation of the imaging findings was only available in a small fraction of cases. Clinical follow-up as a substitute for pathological correlation has its limitations. Thirdly, the study group was not homogeneous. The largest group consisted of 36 patients with HCC while the 24 patients with metastatic disease were fragmented to small subgroups of different primary tumors and in the cholangiocarcinoma group there were only seven lesions. Nevertheless, follow-up showed enhancement at the delayed/portal venous phase imaging in all cases (all also showing partial response) after treatment which seems to result from radiation injury rather than tumor recurrence. Consequently, prospective studies of lesions treated with stereotactic radiotherapy with a larger study group, specifically including cholangiocarcinoma, are needed. Lastly, we have not identified any case of acute or late toxicity. It is possible that we have missed some cases of late toxicity due to retrospective nature of this study. Furthermore, our cyberknife center serves as a referral center for a large area and significant number of patients have follow-up and other oncological treatments at other institutions; therefore follow-up is not always available.

In conclusion, following stereotactic body radiotherapy, radiologically, a partial response in size is the most frequent pattern seen in focal liver lesions, with continuous decrease in volume up to nine months after treatment, while lesion enhancement decreases immediately after treatment and does not change unless recurrence occurs. A prominent halo of delayed and portal venous enhancement of the liver adjacent to the treated lesion is a frequent finding, likely corresponding to postradiation fibrosis.

## Figures and Tables

**Table 1:** Clinical characteristics and treatment response by the different groups of treated lesions.

	Hepatocellular carcinoma	Metastases	Cholangiocarcinoma
*N* of patients/*N* of lesions	32/36	20/24	7/7
Age (yrs)	68 ± 13	68 ± 12	66 ± 11
Male to female ratio	27 (84%) : 5 (16%)	14 (70%) : 6 (30%)	4 (57%) : 3 (43%)
Follow-up time (months)	5.6 ± 7.1	6.4 ± 5.1	10.1 ± 4.8
Response			
Complete response	4 (11%)	1 (4%)	0
Partial response^*∗*^	25 (69%)	14 (58%)	7 (100%)
Partial response followed by recurrence^*∗∗*^	2 (6%)	4 (17%)	0
No response	5 (14%)	5 (21%)	0
Liver parenchyma surrounding the treated lesion			
Hyperdense	27 (75%)	21 (88%)	7 (100%)
Isodense	8 (22%)	3 (12%)	0
Hypodense	1 (3%)	0	0

^*∗*^30% decrease in long diameter.

^*∗∗*^20% increase in long diameter from nadir.

## References

[1] Elaimy A. L., MacKay A. R., Lamoreaux W. T. (2011). Clinical outcomes of stereotactic radiosurgery in the treatment of patients with metastatic brain tumors. *World Neurosurgery*.

[2] Small R., Lubezky N., Ben-Haim M. (2007). Current controversies in the surgical management of colorectal cancer metastases to the liver. *Israel Medical Association Journal*.

[3] Tinkle C. L., Haas-Kogan D. (2012). Hepatocellular carcinoma: natural history, current management, and emerging tools. *Biologics*.

[4] Goyal K., Einstein D., Yao M. (2010). Cyberknife stereotactic body radiation therapy for nonresectable tumors of the liver: preliminary results. *HPB Surgery*.

[5] Louis C., Dewas S., Mirabel X. (2010). Stereotactic radiotherapy of hepatocellular carcinoma: preliminary results. *Technology in Cancer Research & Treatment*.

[6] Janoray G., Barillot I., Calais G. (2014). Evaluation of therapeutic response after stereotactic body radiation therapy for liver tumours. *Cancer/Radiotherapie*.

[7] Kim M.-S., Kang J.-K., Chul K. C. (2009). Three-fraction stereotactic body radiation therapy for isolated liver recurrence from colorectal cancer. *Tumori*.

[8] Rusthoven K. E., Kavanagh B. D., Cardenes H. (2009). Multi-institutional phase I/II trial of stereotactic body radiation therapy for liver metastases. *Journal of Clinical Oncology*.

[9] Dawood O., Mahadevan A., Goodman K. A. (2009). Stereotactic body radiation therapy for liver metastases. *European Journal of Cancer*.

[10] Kress M.-A. S., Collins B. T., Collins S. P., Dritschilo A., Gagnon G., Unger K. (2012). Stereotactic body radiation therapy for liver metastases from colorectal cancer: analysis of safety, feasibility, and early outcomes. *Frontiers in Oncology*.

[11] Eriguchi T., Takeda A., Sanuki N. (2013). Acceptable toxicity after stereotactic body radiation therapy for liver tumors adjacent to the central biliary system. *International Journal of Radiation Oncology Biology Physics*.

[12] Tanguturi S. K., Wo J. Y., Zhu A. X., Dawson L. A., Hong T. S. (2014). Radiation therapy for liver tumors: ready for inclusion in guidelines?. *Oncologist*.

[13] Lencioni R., Llovet J. M. (2010). Modified recist (mRECIST) assessment for hepatocellular carcinoma. *Seminars in Liver Disease*.

[14] Boda-Heggemann J., Dinter D., Weiss C. (2012). Hypofractionated image-guided breath-hold SABR (stereotactic ablative body radiotherapy) of liver metastases—clinical results. *Radiation Oncology*.

[15] Herfarth K. K., Debus J., Lohr F. (2001). Stereotactic single-dose radiation therapy of liver tumors: results of a phase I/II trial. *Journal of Clinical Oncology*.

[16] Wulf J., Hädinger U., Oppitz U., Thiele W., Ness-Dourdoumas R., Flentje M. (2001). Stereotactic radiotherapy of targets in the lung and liver. *Strahlentherapie und Onkologie*.

[17] Andratschke N. H., Nieder C., Heppt F., Molls M., Zimmermann F. (2015). Stereotactic radiation therapy for liver metastases: factors affecting local control and survival. *Radiation Oncology*.

[18] Boda-Heggemann J., Frauenfeld A., Weiss C. (2014). Clinical outcome of hypofractionated breath-hold image-guided SABR of primary lung tumors and lung metastases. *Radiation Oncology*.

[19] Peiffert D., Baumann A.-S., Marchesi V. (2014). Treatment of hepatic metastases of colorectal cancer by robotic stereotactic radiation (Cyberknife). *Journal of Visceral Surgery*.

[20] Andolino D. L., Johnson C. S., Maluccio M. (2011). Stereotactic body radiotherapy for primary hepatocellular carcinoma. *International Journal of Radiation Oncology Biology Physics*.

[21] Iwata H., Shibamoto Y., Hashizume C. (2010). Hypofractionated stereotactic body radiotherapy for primary and metastatic liver tumors using the novalis image-guided system: preliminary results regarding efficacy and toxicity. *Technology in Cancer Research and Treatment*.

[22] Takeda A., Sanuki N., Eriguchi T. (2014). Stereotactic ablative body radiotherapy for previously untreated solitary hepatocellular carcinoma. *Journal of Gastroenterology and Hepatology*.

[23] Faria S. C., Ganesan K., Mwangi I. (2009). MR imaging of liver fibrosis: current state of the art. *Radiographics*.

[24] Ingold J. A., Reed G. B., Kaplan H. S., Bagshaw M. A. (1965). Radiation hepatitis. *The American Journal of Roentgenology, Radium Therapy and Nuclear Medicine*.

[25] Yamasaki S. A., Marn C. S., Francis I. R., Robertson J. M., Lawrence T. S. (1995). High-dose localized radiation therapy for treatment of hepatic malignant tumors: CT findings and their relation to radiation hepatitis. *American Journal of Roentgenology*.

[26] Herfarth K. K., Hof H., Bahner M. L. (2003). Assessment of focal liver reaction by multiphasic CT after stereotactic single-dose radiotherapy of liver tumors. *International Journal of Radiation Oncology, Biology, Physics*.

[27] Maturen K. E., Feng M. U., Wasnik A. P. (2013). Imaging effects of radiation therapy in the abdomen and pelvis: evaluating ‘innocent bystander’ tissues. *Radiographics*.

[28] Lewin K., Millis R. R. (1973). Human radiation hepatitis. A morphologic study with emphasis on the late changes. *Archives of Pathology and Laboratory Medicine*.

